# Effects of different preceding crops on soil properties and rhizosphere microbial community of sugar beet

**DOI:** 10.3389/fpls.2025.1626870

**Published:** 2025-10-21

**Authors:** Yinghao Li, Chunliu Yuan, Chunyan Huang, Zhi Li, Huimin Ren, Peng Zhang, Caiyuan Jian, Kang Han, Dejuan Kong, Zhenzhen Wang, Xiaoxia Guo, Lu Tian

**Affiliations:** ^1^ Inner Mongolia Academy of Agricultural and Animal Husbandry Sciences, Hohhot, China; ^2^ Agriculture and Forestry Sciences of Ulanqab, Jining, China

**Keywords:** sugar beet, preceding crop, soil properties, microbial diversity, community structure

## Abstract

A significant challenge in sugar beet cultivation is the issue of continuous cropping cycles. The implementation of preceding crop cultivation has emerged as an effective strategy to mitigate the problems associated with continuous cropping systems. This study investigates how different preceding crops influence soil properties, microbial diversity, and community structure in the sugar beet rhizosphere, thereby establishing a theoretical foundation for addressing continuous cropping obstacles in sugar beet production. This study utilized a field experiment with four distinct preceding crop treatments-potato, oat, corn, and sunflower-prior to sugar beet cultivation. Subsequent analyses focused on sugar beet growth performance, soil chemical properties, and shifts in microbial community structure. The findings demonstrate that preceding crops significantly alter nutrient availability in sugar beet rhizosphere soil, microbial diversity, and overall crop productivity. Specifically, oat and potato stubbles substantially enhanced soil organic matter content, available nitrogen, phosphorus, and potassium levels, along with increased activities of alkaline phosphatase, urease, and sucrase, ultimately promoting sugar beet growth. Sunflower stubble exhibited distinct effects, notably increasing bacterial diversity while reducing fungal diversity. Across all treatments, the dominant bacterial phyla in the sugar beet rhizosphere were Firmicutes and Acidobacteria, whereas Ascomycota and Mortierellomycota prevailed among fungal communities. Importantly, sunflower stubble exerted the most pronounced influence on the relative abundance of these dominant bacterial and fungal phyla.

## 1 Introduction

Sugar beet (Beta vulgaris L.) is not only a vital global feed source but also a key raw material for the sugar industry (Chhikara et al., 2019; Li et al., 2020). Accounting for approximately one-third of the global sugar crop cultivation area, sugar beet contributes to 16% of the world’s sugar production (Geng and Yang, 2015). In terms of planting area and output, only sugarcane surpasses sugar beet (Mall et al., 2021). As a taproot crop, sugar beet is unsuitable for continuous monocropping. A healthy soil environment is essential for achieving high yields (Geng et al., 2020; Huang et al., 2021). However, improper land use and cultivation practices have led to widespread continuous cropping of sugar beet, resulting in nutrient imbalances, shifts in soil microbial communities, loss of soil organic matter, increased susceptibility to crop diseases, and reduced sugar content and yield (Huang et al., 2020). Factors such as limited arable land resources, inadequate farming management systems, and environmental constraints (Deihimfard et al., 2019; Holmquist et al., 2021) have exacerbated the challenges associated with continuous cropping, making this an urgent issue to address. Since sugar beet is a cross-pollinated crop, it is typically cultivated in rotation with cereals. To mitigate the adverse effects of continuous cropping, we have conducted experiments involving the cultivation of different crops during the sugar beet fallow period.

Preceding crop cultivation is a widely adopted agricultural practice aimed at mitigating crop damage caused by soil-borne pathogens. This technique effectively reduces the likelihood of pathogen invasion into plant roots by creating unfavorable conditions for their growth (Kang et al., 2020). The strategic selection of appropriate preceding crops offers multiple benefits, including enhanced nutrient cycling, increased enzymatic activity, stabilization of microbial community structure and functional diversity (Trinchera et al., 2022), optimization of rhizosphere metabolite composition and abundance, and improvement of soil metabolic pathways (Wang et al., 2023). Consequently, careful consideration of preceding crops presents an effective solution to the challenges associated with continuous cropping systems (Qin et al., 2017). Extensive research has demonstrated the significant influence of preceding crops on soil microbial composition and population dynamics. For example, maize-based crop rotations have been shown to elevate the relative abundance of specific beneficial soil bacteria while simultaneously improving carbon, nitrogen, and phosphorus cycling (De Graaff et al., 2010). Similarly, rapeseed cultivation has been found to enhance microbial diversity and foster symbiotic relationships among species in rice rhizosphere soils (Zhang et al., 2023). Furthermore, wheat as a preceding crop has been observed to support rhizosphere-derived bacterial communities in subsequent soybean crops, thereby providing protection against soil-borne diseases (Yin et al., 2023). Soil microorganisms play a pivotal role in ecosystem functioning, particularly in the cycling of essential nutrients such as carbon, nitrogen, and phosphorus (De Graaff et al., 2010). These microbial communities contribute to plant growth promotion through organic matter decomposition and the enhancement of plant defense mechanisms against pathogens (Adekunle et al., 2021). The strategic implementation of preceding crop systems therefore represents a comprehensive approach to sustainable soil management and crop protection.

To date, the regulation of rhizosphere soil microbial community structure through preceding crops has not been sufficiently studied. Therefore, this study aims to modulate the soil properties, microbial diversity, and community structure in the sugar beet rhizosphere by planting different preceding crops (sunflower, maize, oat, and sunflower) and proposes the following hypotheses: (1) Preceding crops recruit specific microbial populations, thereby shaping the microbial community structure and function in the sugar beet rhizosphere; (2) Preceding crops enhance soil nutrient content by regulating the microbial community structure in the sugar beet rhizosphere. The objective of this study is to identify suitable preceding crops for sugar beet cultivation by analyzing soil properties and rhizosphere microorganisms, providing a theoretical basis for balancing the soil environment in the sugar beet rhizosphere and addressing the challenges associated with continuous sugar beet cropping.

## 2 Materials and methods

### 2.1 Site description and test materials

This experiment was conducted in 2022 at the experimental field of the Agricultural and Animal Husbandry Science Research Institute located in Ulanqab, Inner Mongolia Autonomous Region. The test site is situated in Quan Town (40.9232°N, 113.1196°E), Tiding Ground, Chaiyouqian Banner, Wulanchapu City, Inner Mongolia Autonomous Region. The region exhibits a mid-temperate continental monsoon climate characterized by cold and dry conditions, frequent winds, scarce rainfall, and significant diurnal temperature variation. The average annual temperature is 4.5°C, with extremes ranging from a high of 39.7°C to a low of -34.4°C. Annual precipitation totals 376.1 mm, predominantly occurring from July to early August. The average annual frost-free period lasts 131 days. The soil type is classified as typical chestnut soil, with an organic matter content of 18.21 g·kg^-1^, total nitrogen content of 0.71 g·kg^-1^, total phosphorus content of 0.46 g·kg^-1^, total potassium content of 16.31 g·kg^-1^, alkaline hydrolyzable nitrogen content of 111.07 mg·kg^-1^, available phosphorus content of 9.23 mg·kg^-1^, and available potassium content of 153.01 mg·kg^-1^. The previous crop of the sugar beet experimental field was formed by simultaneously planting four crops-potatoes, oats, corn and sunflowers - on the zucchini plot in 2021, resulting in four different crop rotations.

The tested beet variety was IM1162, and the fertilizer used for beet cultivation was selected as (12-18-15), with a total nutrient content of at least 40%.

### 2.2 Experimental design

This study employed a large-plot experimental design to investigate four distinct preceding crop treatments: potato stubble (P), oat stubble (O), corn stubble (C), and sunflower stubble (S), each with three replicates. Each treatment plot covered an area of 200 m^2^, with uniform cultivation practices implemented across all experimental units. Sugar beet seedlings were cultivated in paper pots starting April 10th and subsequently transplanted on May 25th. The planting configuration maintained 50 cm row spacing and 25 cm plant spacing, achieving a theoretical planting density of 8.0×104 plants·hm^-2^.

Prior to planting, a basal application of sugar beet-specific compound fertilizer (NPK ratio 12-18-15) was incorporated into the soil at a rate of 900 kg·hm^-2^ through tillage operations. Drip irrigation systems were employed throughout the growth cycle to maintain optimal soil moisture levels for crop development. All other agronomic management practices followed conventional field production protocols.

### 2.3 Soil collection

Following sugar beet harvest, three biological replicates were obtained through random sampling of three spatially distributed points within experimental plots. Sampling focused on the agriculturally critical topsoil layer (0–20 cm depth), which maintained 80% of the root biomass and the related rhizosphere microbiota of sugar beet. The collected soil was homogenized and sieved through a 2 mm mesh to ensure particle size consistency. Processed samples were subsequently subjected to DNA extraction followed by high-throughput sequencing analysis to characterize microbial communities.

### 2.4 Determination of soil chemical properties and enzyme activities

The chemical properties of soil included organic matter content, alkali-hydrolyzed nitrogen content, available phosphorus content and available potassium content. The enzyme activities of soil included alkaline phosphatase activity, urease activity, and sucrase activity. The analysis of all indexes was based on Liu R. H. et al. (2020).

### 2.5 DNA extraction, PCR quantification, library preparation and sequencing

All soil specimens were cryopreserved at -80°C prior to analysis. Total genome DNA from samples was extracted using CTAB/SDS method. Genomic DNA extraction from 250 mg soil aliquots was performed utilizing the HiPure Soil DNA Kit (Magen, China) following the manufacturer’s protocol. Quantitative analysis of 16S rRNA (bacterial) and ITS (fungal) gene abundance was conducted using an iQ5 Real-Time PCR Detection System (Bio-Rad Laboratories, Hercules, CA, USA) with the following primer sets: - Bacterial communities: 515F (5’-GTGCCAGCMGCCGCGGTAA -3’) and 806R (5’-GGACTACHVGGGTWTCTAAT -3’)- Fungal communities: ITS1-1F-F (5’-CTTGGTCATTTAGAGGAAGTAA -3’) and ITS1-1F-R (5’-GCTGCGTTCTTCATCGATGC -3’). PCR products were mixed with 1X loading buffer containing SYBR Green, then separated by electrophoresis on a 2% agarose gel. PCR products was mixed in equidensity ratios. Then, mixture PCR products was purified with Qiagen Gel Extraction Kit(Qiagen, Germany). Library preparation and sequencing libraries were generated using Illumina TruSeq DNA PCR-Free Library Preparation Kit (Illumina, USA) following manufacturer’s recommendations and index codes were added. The library quality was assessed on the Qubit@ 2.0 Fluorometer (Thermo Scientific) and Agilent Bioanalyzer 2100 system. At last, the library was sequenced on an Illumina NovaSeq platform and 250 bp paired-end reads were generated.

### 2.6 Analysis of soil microbial diversity

All statistical analyses were conducted using SPSS 23.0 software (IBM Corporation) with a significance threshold set at p< 0.05. Initial data processing employed Tukey’s Honestly Significant Difference (HSD) *post-hoc* test for multiple comparisons, followed by Pearson correlation analysis to assess variable associations. Graphical representations of results were generated using GraphPad Prism version 8.00 (GraphPad Software) for enhanced visual clarity. Microbial community analysis was performed through Quantitative Insights Into Microbial Ecology (QIIME) pipeline version 1.9.0. Taxonomic sequencing data underwent rigorous processing including quality filtering, operational taxonomic unit (OTU) clustering, and diversity calculations. Community structure visualization was achieved through principal coordinates analysis (PCoA) based on Bray-Curtis dissimilarity matrices, enabling effective comparison of bacterial and fungal assemblages across treatment groups. Advanced ecological analyses were implemented in R Studio (R Foundation for Statistical Computing) using the vegan package (version 2.5-6). This included comprehensive beta diversity assessments coupled with redundancy analysis (RDA) and Mantel tests to evaluate environmental factor influences on microbial community composition. All multivariate statistics maintained consistent significance thresholds (α = 0.05) throughout the analytical workflow.

### 2.7 Dry matter accumulation, yield and sugar content

Samples were collected at harvest stage. Samples (n = 3) were randomly selected at 3 points in each plot, among which 3 plants were selected at each point. Samples were brought back to the laboratory for green removal at 105 °C for 30 min, and then dried at 80°C until reaching a constant weight.

To calculate root yield, 10 m^2^ area were chosen in a plot, green tops of plants were cut, roots were removed and clean from soil around roots. All beets were weighed separately for each plot. In order to measure the root brix, 15 sugar beet roots were chosen at random from a plot and brix reading was measured through a Japanese-made Atago Refractometer PAL-1 digital handheld refractometer, and sugar content was converted. The sugar content and sugar yield were calculated as shown below.


Sugar Content = The root brix × 80%

Sugar Yield =Root yield × Sugar content

### 2.8 Data analysis

In this study, the charts were made using Microsoft Excel 2021 software. All data were expressed as the mean ± SE. One-way ANOVA was performed to test the significance of the observed differences using SPSS (Inc., Chicago, USA). Differences between parameters were evaluated using Duncan’ s method, and P ≤ 0.05 was considered the statistically significant threshold.

## 3 Results

### 3.1 Analysis of soil chemical indexes under different treatments

The soil organic matter content, available nitrogen content, available phosphorus content, available potassium content, alkaline phosphatase activity, urease activity, and sucrase activity generally exhibited the trend of O>P>C>S among the treatments (**Table 1**). No significant differences were observed between treatments O and P in any of the measured indicators, but both O and P were significantly higher than C and S in all parameters. Compared with treatment C, all indicators in O and P increased by 9.52% and 5.25%, 13.30% and 11.30%, 11.84% and 10.09%, 11.17% and 9.55%, 7.34% and 5.81%, 25.29% and 19.01%, and 7.86% and 5.93%, respectively (P ≤ 0.05). Compared with treatment S, these indicators in O and P increased by 16.96% and 12.39%, 18.55% and 16.45%, 25.91% and 23.95%, 14.67% and 13.00%, 13.04% and 11.43%, 33.17% and 26.49%, and 11.98% and 14.03%, respectively (P ≤ 0.05).

**Table 1:** Effects of different treatments on soil chemical properties.

Treatment	Organic matter content (g·kg^-1^)	Alkali-hydrolyzed nitrogen content (mg·kg^-1^)	Available phosphorus content (mg·kg^-1^)	Available potassium content (mg·kg^-1^)	Alkaline phosphatase activity (mg·g^-1^·24h^-1^)	Urease activity (mg·g^-1^·24h^-1^)	Sucrase activity (mg·g^-1^·24h^-1^)
S	15.66 ± 0.26 c	119.00 ± 0.64 c	5.85 ± 0.11 c	119.09 ± 1.18 c	7.80 ± 0.24 c	0.40 ± 0.01 b	11.40 ± 0.11 b
C	16.72 ± 0.12 b	124.51 ± 2.53 b	6.59 ± 0.06 b	122.85 ± 0.49 b	8.21 ± 0.26 b	0.43 ± 0.02 b	12.05 ± 0.18 b
O	18.31 ± 0.69 a	141.07 ± 1.40 a	7.37 ± 0.20 a	136.56 ± 2.34 a	8.82 ± 0.09 a	0.53 ± 0.04 a	12.99 ± 0.12 a
P	17.60 ± 0.54 a	138.57 ± 2.02 a	7.25 ± 0.09 a	134.57 ± 1.02 a	8.69 ± 0.30 a	0.51 ± 0.02 a	12.76 ± 0.45 a

S, C, O, and P represent sunflower stubble, corn stubble, oat stubble and potato stubble, respectively. Different letters are signiﬁcantly different (P<0.05, Tukey’s HSD test).

### 3.2 Alpha diversities of bacterial and fungal communities in soil

Compared with sunflower stubble (S), the corn stubble (C), oat stubble (O), and potato stubble (P) signifcantly affected the alpha diversity index of soil bacteria (**Table 2**). Compared with treatment S, the soil bacterial observed_species index and ACE index in treatment C were significantly reduced by 8.52% and 13.33%, respectively. In treatment O, the soil bacterial observed_species index, shannon index, and simpson index were significantly decreased by 10.58%, 7.05%, and 0.40%, respectively. In treatment P, the soil bacterial observed_species index, shannon index, simpson index, and ACE index were significantly reduced by 12.82%, 7.16%, 0.40%, and 13.30%, respectively (P ≤ 0.05). Compared with treatment C, treatment S signifcantly affected the alpha diversity index of soil fungi. Compared with treatment C, the soil fungal shannon index and simpson index in treatment S were significantly reduced by 12.59% and 5.03%, respectively (P ≤ 0.05).

**Table 2:** Variance analysis of alpha diversity index of soil bacteria and fungi under different preceding crops.

Microorganisms	Treatment	Observed_species	Shannon	Simpson	Chao1	ACE	Goods_coverage
Bacteria	S	4858 ± 206.18 a	10.505 ± 0.117 a	0.998 ± 0.001 a	6488.05 ± 423.11 a	6637.59 ± 383.43 a	0.959 ± 0.003 a
	C	4444 ± 348.49 b	10.156 ± 0.317 a	0.997 ± 0.001 a	5591.50 ± 840.03 a	5753.13 ± 741.13 b	0.966 ± 0.007 a
	O	4344 ± 138.16 b	9.764 ± 0.183 b	0.994 ± 0.002 b	5817.37 ± 319.80 a	6017.19 ± 276.99 ab	0.962 ± 0.002 a
	P	4235 ± 95.30 b	9.753 ± 0.054 b	0.994 ± 0.001 b	5585.26 ± 248.81 a	5754.82 ± 241.42 b	0.964 ± 0.002 a
Fungi	S	720 ± 254.81 a	5.568 ± 0.396 b	0.925 ± 0.024 b	997.12 ± 470.41 a	966.43 ± 437.60 a	0.995 ± 0.003 a
	C	897 ± 74.04 a	6.370 ± 0.016 a	0.974 ± 0.002 a	1140.67 ± 149.88 a	1159.19 ± 151.09 a	0.994 ± 0.001 a
	O	774 ± 78.34 a	5.924 ± 0.169 ab	0.959 ± 0.005 a	1012.26 ± 170.44 a	1036.51 ± 160.36 a	0.995 ± 0.001 a
	P	750 ± 70.79 a	5.920 ± 0.218 ab	0.961 ± 0.014 a	957.03 ± 86.12 a	974.37 ± 110.45 a	0.995 ± 0.001 a

S, C, O, and P represent sunflower stubble, corn stubble, oat stubble and potato stubble, respectively. Different letters are signiﬁcantly different (P<0.05, Tukey’s HSD test).

### 3.3 Beta diversities of bacterial and fungal communities in soil

**Figure 1:**
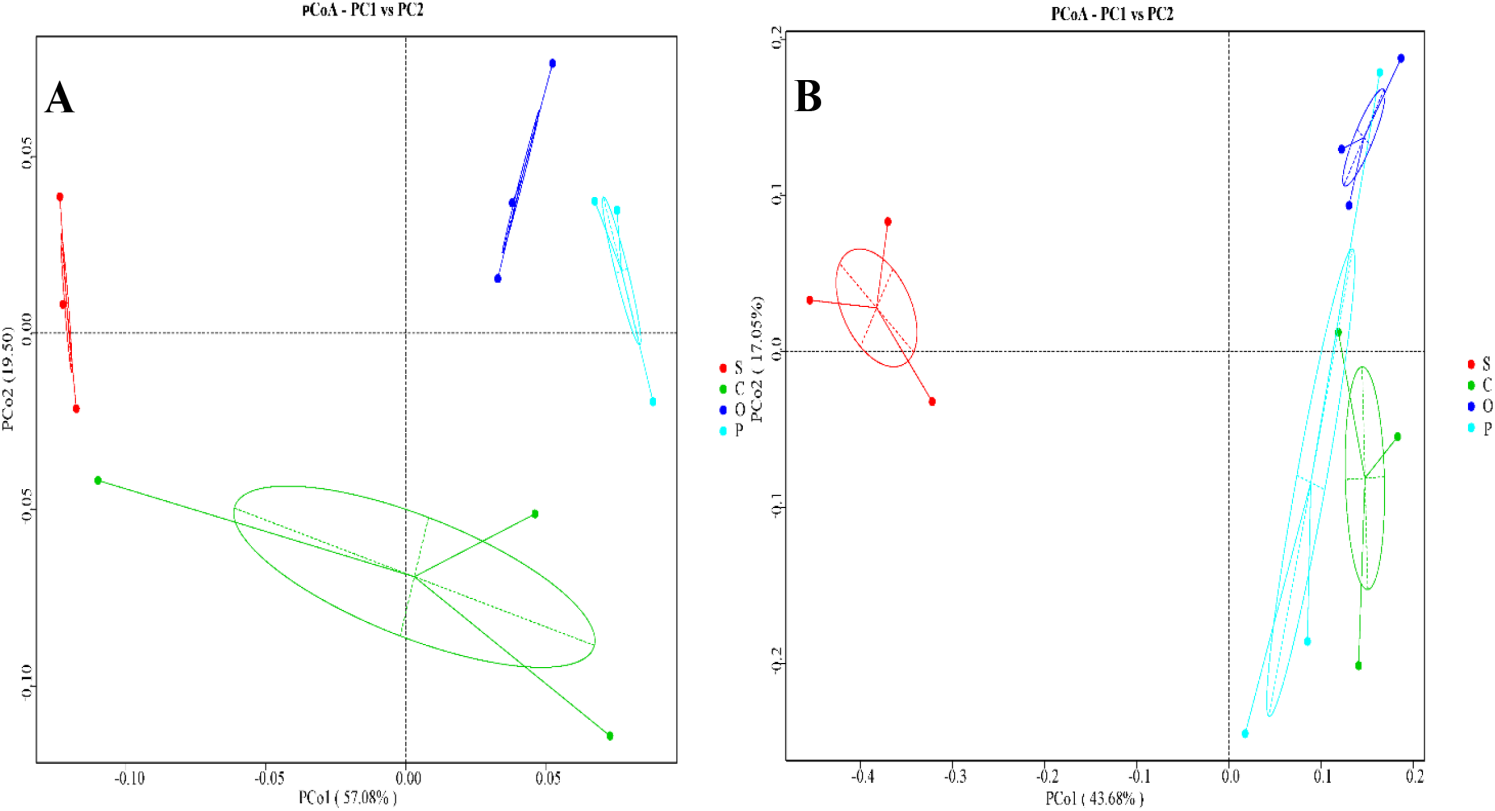
Principal coordinate analysis (PCoA) of bacterial **(A)** and fungal **(B)** microbial communities in the rhizosphere soil of sugar beet under different treatments. Beta diversities based on Bray Curtis distance dissimilarity were visu-alized by principal component analyses. OTUs were delineated at 97% sequence similarity. S, C, O, and P represent sunflower stubble, corn stubble, oat stubble and potato stubble, respectively.

In the bacterial community of sugar beet rhizosphere soil, Principal Coordinates Analysis (PCoA) revealed that PCoA1 and PCoA2 axes explained 57.08% and 19.50% of the compositional variations, respectively. The S, C, O, and P treatments formed distinct clusters, indicating independent community structures (**Figure 1A**). The C, O, and P groups were all distant from S, with no overlap observed among the different treatments, suggesting significant differences and dissimilarities in community composition. Notably, the distance between P and S was the greatest, implying substantial differences in soil bacterial composition and community structure between these two treatments. In the fungal community of sugar beet rhizosphere soil, PCoA showed that PCoA1 and PCoA2 axes accounted for 43.68% and 17.05% of the compositional variations, respectively. Similarly, the S, C, O, and P treatments formed separate clusters (**Figure 1B**). While C, O, and P were all distant from S, partial overlaps were observed among the C, O, and P clusters, indicating relatively minor differences in fungal operational taxonomic unit (OTU) composition and partially similar fungal community structures among these three treatments.

### 3.4 Composition and relative abundance of bacterials and fungal communities in soil

**Figure 2:**
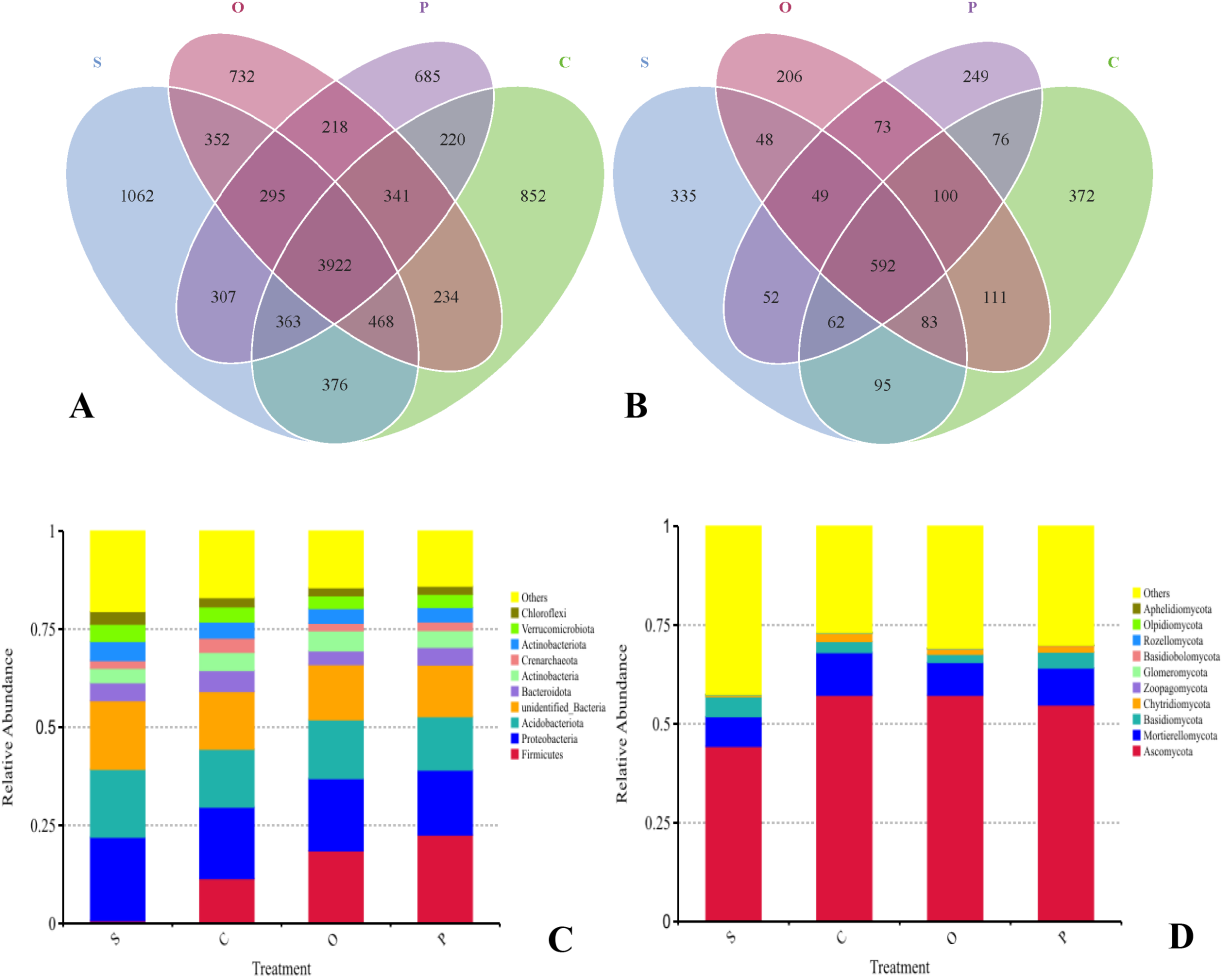
Venn diagram analyses of bacterial **(A)** and fungi **(B)** and relative abundances of bacterial **(C)** and fungal **(D)** phyla and in soil of different treatments. Bacterial and fungal phyla with average relative abundance >1% are shown and do not contain unclassified taxa. Data are represented as the means of three independent replicates. Venn diagram **(C, D)** demonstrates the numbers of shared and unique observed OTUs at 97% similarity among treatments. S, C, O, and P represent sunflower stubble, corn stubble, oat stubble and potato stubble, respectively.

The rhizosphere soil of sugar beets harbored a substantial number of shared bacterial OTUs, totaling 10,427. Among the different treatments, the number of unique bacterial species followed the order: S>C>O>P (**Figure 2A**; **Supplementary Table S1**). Specifically, treatment S exhibited the highest count of unique bacterial OTUs (7,145), whereas treatment P had the lowest (6,351). In contrast, fungal OTUs displayed a different pattern, with 2,503 shared species across treatments. The abundance of unique fungal species was ranked as C>S>O>P (**Figure 2B**; **Supplementary Table S2**). Treatment C contained the highest number of unique fungal OTUs (1,491), while treatment P had the fewest (1,253).

The rhizosphere soil microbial community structure of sugar beets exhibited distinct hierarchical layers. The dominant bacterial phyla included Firmicutes (C: 0.69%; C: 11.42%; O: 18.52%; P: 22.57%), Proteobacteria (C: 21.29%; C: 18.23%; O: 18.43%; P: 16.51%), and Acidobacteriota (C: 17.32%; C: 14.76%; O: 14.98%; P: 13.63%), collectively accounting for approximately 53.19%-74.46% (**Figure 2C**). In addition to the dominant bacterial phyla, others such as Bacteroidota (C: 4.60%; C: 5.35%; O: 3.49%; P: 4.48%), Actinobacteria (C: 3.66%; C: 4.64%; O: 5.16%; P: 4.31%), Crenarchaeota (C: 1.98%; C: 3.62%; O: 1.90%; P: 2.20%), Actinobacteriota (C: 4.89%; C: 4.12%; O: 3.76%; P: 3.69%), Verrucomicrobiota (C: 4.39%; C: 3.85%; O: 3.25%; P: 3.36%), and Chloroflexi (C: 3.22%; C: 2.35%; O: 2.07%; P: 2.05%) also showed varying degrees of response to soil cover treatments (**Figure 2C**; **Supplementary Table S3**). The dominant fungal phyla in sugar beet rhizosphere soil were Ascomycota (C: 44.31%; C: 57.27%; O: 57.27%; P: 54.77%) and Mortierellomycota (C: 7.50%; C: 10.76%; O: 8.29%; P: 9.34%), together accounting for approximately 35.88%-213.62% (**Figure 2D**). Besides these dominant fungal phyla, others such as Basidiomycota (C: 5.07%; C: 2.83%; O: 2.07%; P: 4.05%) and Chytridiomycota (C: 0.42%; C: 2.04%; O: 1.31%; P: 1.62%) also exhibited some degree of response to soil cover treatments (**Figure 2D**; **Supplementary Table S4**).

### 3.5 The response of different species to various treatments in soil

Microbial community analysis of sugar beet rhizosphere soil revealed 14 bacterial phyla with a relative abundance exceeding 0.5% (**Table 3**). The most dominant phyla included Proteobacteria, Acidobacteriota, Planctomycetota, Chloroflexi, Actinobacteriota, Verrucomicrobiota, Planctomycetes, Gemmatimonadota, Myxococcota, and Methylomirabilota, with treatment S exhibiting the highest relative abundances for these taxa. In contrast, Firmicutes consistently displayed the lowest abundance in treatment S. Notably, treatment C was distinguished by a significantly greater relative abundance of Bacteroidota and Crenarchaeota compared to the other treatments. Fungal community analysis identified only three phyla with a relative abundance above 0.5% (**Table 4**). Ascomycota and Mortierellomycota emerged as the dominant fungal phyla, reaching their highest abundances in treatment C. Conversely, treatment S demonstrated a substantially greater relative abundance of Basidiomycota than the other treatments.

**Table 3:** Relative abundances of dominant bacterial phyla in soil.

Taxonomy	S	C	O	P
Proteobacteria	21.28 ± 0.34 a	18.20 ± 1.70 b	18.47 ± 1.24 b	16.50 ± 1.33 b
Actinobacteria	3.66 ± 0.24 b	4.63 ± 0.87 ab	5.17 ± 0.91 a	4.30 ± 0.58 ab
Firmicutes	0.69 ± 0.08 c	11.41 ± 9.02 b	18.54 ± 4.05 ab	22.58 ± 0.98 a
Acidobacteriota	17.34 ± 1.99 a	14.77 ± 2.08 ab	14.98 ± 0.66 ab	13.63 ± 1.55 b
Bacteroidota	4.60 ± 0.70 ab	5.37 ± 0.22 a	3.49 ± 0.33 b	4.48 ± 1.31 ab
Crenarchaeota	1.98 ± 0.40 ab	3.62 ± 1.67 a	1.90 ± 0.31 b	2.20 ± 0.19 ab
Planctomycetota	3.03 ± 0.11 a	1.88 ± 0.80 b	1.64 ± 0.15 b	1.54 ± 0.25 b
Chloroflexi	3.23 ± 0.18 a	2.32 ± 0.67 b	2.07 ± 0.17 b	2.03 ± 0.12 b
Actinobacteriota	4.91 ± 0.13 a	4.14 ± 0.79 ab	3.75 ± 0.29 b	3.69 ± 0.48 b
Verrucomicrobiota	4.39 ± 0.09 a	3.83 ± 0.49 b	3.24 ± 0.16 c	3.37 ± 0.14 bc
Planctomycetes	1.09 ± 0.09 a	0.61 ± 0.30 b	0.60 ± 0.03 b	0.60 ± 0.08 b
Gemmatimonadota	2.02 ± 0.26 a	1.40 ± 0.33 b	1.14 ± 0.06 b	1.18 ± 0.05 b
Myxococcota	1.89 ± 0.17 a	1.73 ± 0.24 a	1.63 ± 0.16 ab	1.40 ± 0.11 b
Methylomirabilota	1.01 ± 0.15 a	0.93 ± 0.32 a	0.84 ± 0.09 a	0.83 ± 0.01 a

Different letters are signiﬁcantly different (p< 0.05, Tukey’s HSD test). S, C, O, and P represent sunflower stubble, corn stubble, oat stubble and potato stubble, respectively.

**Table 4:** Relative abundances of dominant fungal genus in soil.

Taxonomy	S	C	O	P
Ascomycota	44.32 ± 6.81 b	57.27 ± 2.32 a	57.26 ± 6.21 a	54.78 ± 5.13 a
Mortierellomycota	7.50 ± 1.93 a	10.76 ± 1.53 a	8.28 ± 1.71 a	9.34 ± 1.81 a
Basidiomycota	5.07 ± 1.81 a	2.83 ± 0.35 bc	2.07 ± 0.33 c	4.04 ± 0.73 ab

Different letters are signiﬁcantly different (p< 0.05, Tukey’s HSD test). S, C, O, and P represent sunflower stubble, corn stubble, oat stubble and potato stubble, respectively.

### 3.6 Relationships between soil microbial communities and soil chemical properties

**Figure 3:**
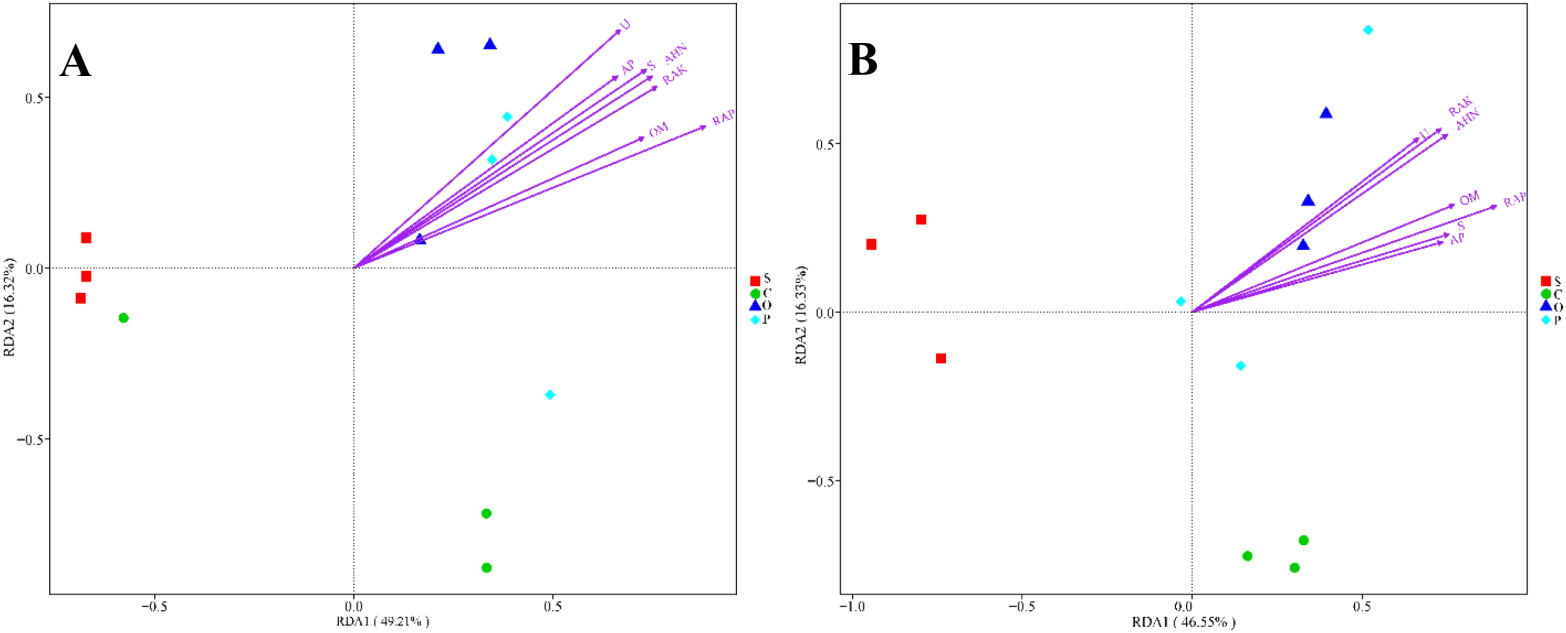
Redundancy analysis (RDA) of soil bacterial **(A)** and fungal **(B)** microbial community structure with soil environmental factors. The environmental variables with statistical significance are presented by arrows. S, C, O, and P represent sunflower stubble, corn stubble, oat stubble and potato stubble, respectively. OM, AHN, RAP, RAK, AP, U, and S represent organic matter, alkali-hydrolyzed nitrogen, available phosphorus, available potassium, alkaline phosphatase, urease and sucrase, respectively.

A redundancy analysis (RDA) was used to analyze the relationship between the changes in the bacterial and fungal community structures and the environmental factors among the different treatments. In RDA1 and RDA2 axes, the explanatory power was 49.21% and 16.32%, respectively (**Figure 3A**). Soil OM, AHN, RAP, RAK, AP, U, and S showed positive correlations with the P and O treatments but negative correlations with the S and C treatments. In RDA1 and RDA2 axes, the explanatory power was 46.55% and 16.33%, respectively (**Figure 3B**). Soil OM, AHN, RAP, RAK, AP, U, and S exhibited positive correlations with the O treatment but negative correlations with the S and C treatments.

### 1.7 Effects of different treatments on growth of sugar beet

**Figure 4:**
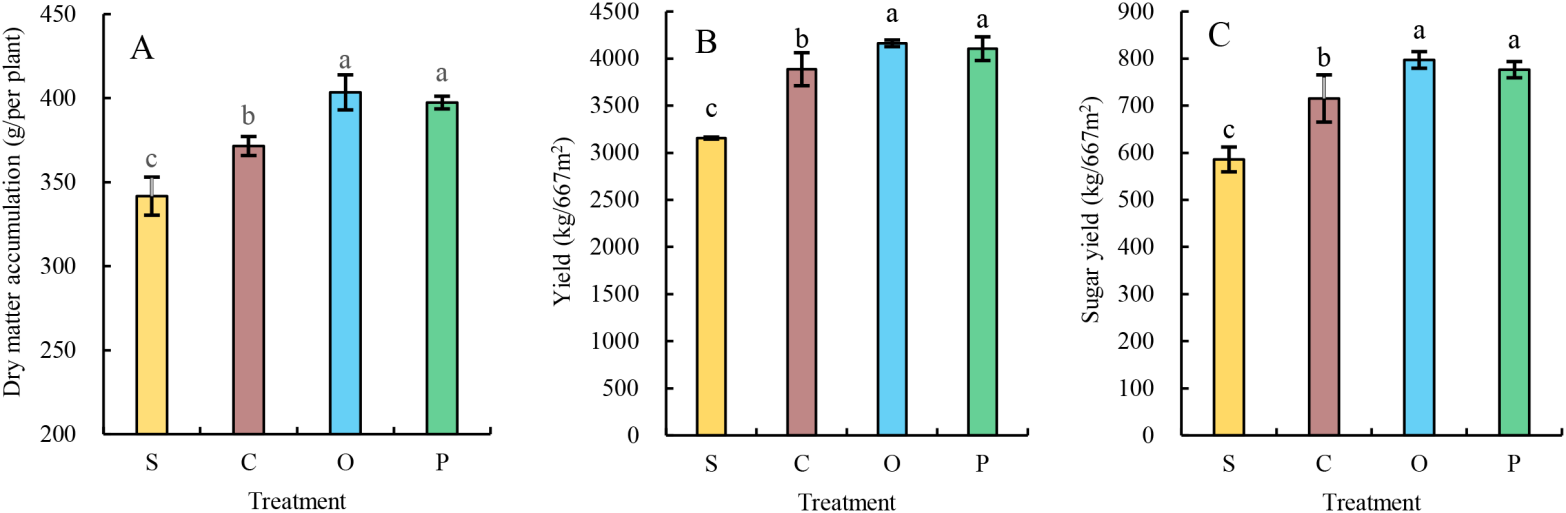
Effects of different treatments on dry matter accumulation **(A)**, yield **(B)**, and sugar yield **(C)** of Sugar Beet. Different lower letters indicate significant differences. between processes (P<0.05). S, C, O, and P represent sunflower stubble, corn stubble, oat stubble and potato stubble, respectively. Different letters are significantly different (p<0.05, Tukey’s HSD test).

The single-plant dry matter accumulation (**Figure 4A**), yield (**Figure 4B**), and sugar yield (**Figure 4C**) of sugar beet generally exhibited the trend of O>P>C>S across treatments. No significant differences were observed between O and P treatments in any measured indices, but both O and P treatments were significantly higher than C and S. Compared with treatment C, O and P increased single-plant dry matter accumulation, yield, and sugar yield by 8.60% and 6.96%, 7.07% and 5.61%, 11.44% and 10.09%, and 11.17% and 8.55%, respectively (P ≤ 0.05). Compared with treatment S, O and P enhanced these indices by 18.08% and 16.30%, 31.88% and 30.07%, and 36.06% and 32.52%, respectively (P ≤ 0.05).

## 4 Discussion

Rational crop rotation is an effective solution to address a series of issues caused by continuous cropping obstacles, such as slow growth, stunted plants, and declines in yield and quality. Dry matter accumulation serves as the foundation for sugar beet yield formation. Guo et al. (2016) demonstrated that the effect of different preceding crops on flax dry matter accumulation followed the order: legume crop > buckwheat > millet > potato > no rotation. Zhao et al. (2025) demonstrated that all three preceding crops (corn, rapeseed, wheat, and tobacco) increased tobacco biomass. Zhang et al. (2014) found that the dry matter per plant of wheat was higher after maize than after rice, with significant differences among preceding crops. The results of this experiment indicate significant differences in dry matter accumulation per sugar beet plant across different preceding crops, with potato and oat stubbles significantly outperforming maize and sunflower stubbles. This variation can be attributed to the distinct soil environments created by different preceding crops. Oats, a fibrous-rooted crop, have a well-developed root system, while potatoes, as tuber crops, reduce soil bulk density due to underground tuber growth, resulting in looser soil and higher porosity. These two crops provide a favorable soil environment for subsequent sugar beet growth, thereby promoting its development. In contrast, maize and sunflower stubbles tend to compact the soil, which is less conducive to the growth of subsequent sugar beet crops.

Research has demonstrated that the selection of preceding crops plays a crucial role in promoting plant growth throughout maturity by optimizing nutrient supply (Zhang et al., 2022). Notably, comparative studies have revealed that sugar beet biomass reached its maximum when oats served as the preceding crop, significantly outperforming rotations with potato, maize, and sunflower (Chichongue et al., 2022). This finding underscores the profound influence of preceding crop selection on subsequent planting performance. Soil nutrient availability is a fundamental determinant of agricultural crop productivity (Wulanningtyas et al., 2021; Vaziritabar et al., 2024). In this study, sugar beet rhizosphere soil following oat and potato preceding crops exhibited markedly higher concentrations of available phosphorus and potassium compared to rotations with sunflower and maize. The elevated phosphorus levels may result from excessive phosphorus fertilization practices (Yan et al., 2023), whereas the reduced potassium availability in sunflower- and maize-preceded soils could be attributed to their high potassium uptake efficiency (He et al., 2022). Furthermore, enzymatic activity analyses revealed that urease, alkaline phosphatase, and sucrase activities were significantly enhanced in sugar beet rhizosphere soil following oat and potato preceding crops, with the most pronounced improvement observed under oat cultivation. These enzymatic shifts contributed to improved soil fertility, as indicated by increased levels of alkali-hydrolyzable nitrogen and organic matter in the sugar beet rhizosphere (Borase et al., 2020). Such enhancements in soil biochemical properties create a more favorable growth environment for subsequent crops, thereby optimizing agricultural productivity.

Soil microorganisms are integral to sustainable agriculture, significantly enhancing soil fertility through their involvement in critical biochemical processes, including organic carbon decomposition, humus formation, and the transformation and cycling of essential soil nutrients (Xu et al., 2024). The stability and resilience of microbial communities are largely governed by the modularity of their interactions, which are shaped by multiple ecological factors such as resource allocation, habitat heterogeneity, phylogenetic relationships, and niche overlap (Qiao et al., 2024). Notably, recent research has elucidated the modular architecture of rhizosphere soil microbial networks, demonstrating that their structural organization varies significantly in response to different preceding crops (Liu et al., 2020). This underscores the profound influence of crop rotation history on the compositional diversity of rhizosphere soil microbial communities (Li et al., 2014).

Additionally, predecessor crops can enhance soil bacterial abundance and diversity, thereby promoting crop growth (Xi et al., 2021). In this study, compared with sunflower stubble, the corn stubble, oat stubble, and potato stubble signifcantly affected the alpha diversity index of soil bacteria and fungi (**Table 2**). Compared with sunflower stubble, the soil bacterial observed_species index, and ACE index in corn stubble, the soil bacterial observed_species index, shannon index, and simpson index in oat stubble, and the soil bacterial observed_species index, shannon index, simpson index, and ACE index potato stubble were significantly decreased. In addition, compared with corn stubble, the soil fungal shannon index and simpson index in sunflower stubble were significantly reduced.

Redundancy analysis demonstrated a significant correlation between soil physicochemical properties and soil microbial composition. In this study, we found that soil organic matter content, alkali-hydrolyzed nitrogen content, available phosphorus content, available potassium content, alkaline phosphatase activity, urease activity, and sucrase activity showed positive correlations with the potato stubble and oat stubble but negative correlations with the sunflower stubble and corn stubble; additionally, soil organic matter content, alkali-hydrolyzed nitrogen content, available phosphorus content, available potassium content, alkaline phosphatase activity, urease activity, and sucrase activity exhibited positive correlations with the oat stubble treatment but negative correlations with the sunflower stubble and corn stubble.

Different predecessor crops primarily affect the bacterial phyla as Acidobacteriota, Chloroflexi, and Patescibacteria. Our study obtained similar results: different preceding crops mainly influenced bacterial phyla in sugar beet rhizosphere soil, including Acidobacteriota and Chloroflexi. Acidobacteriota decompose soil plant litter and utilize carbohydrates from root exudates (De Chaves et al., 2019). We also found that when sunflower was used as the preceding crop, the abundance of Chloroflexi in the rhizosphere soil of sugar beet was the highest. Chloroflexi plays a critical role in the decomposition and metabolism of soil organic matter and the establishment of microbial communities (Nabi et al., 2022). For this reason, the soil organic matter content was the lowest in the rhizosphere soil of sugar beet following sunflower as the preceding crop in this study. The Ascomycota group typically accounts for over 90% of fungal species (Wu et al., 2020) and increases significantly after maize cultivation. Surprisingly, this study found that the dominant fungal phylum in the rhizosphere soil of sugar beet under different preceding crop treatments was also Ascomycota (sunflower stubble: 44.31%; corn stubble: 57.27%; oat stubble: 57.27%; potato stubble: 54.77%). Moreover, the richness of Ascomycota in the sugar beet rhizosphere soil was highest when maize was used as the preceding crop. Ascomycota are key decomposers of lignin-rich organic matter and play a crucial role in releasing nutrients essential for plant growth. Root exudates strongly influence the diversity and composition of active fungal populations (Ji et al., 2022). Different preceding crops alter the microbial community in the sugar beet rhizosphere, which may affect plant-microbe interactions and nutrient cycling, thereby promoting sugar beet growth.

## 5 Conclusion

Overall, preceding crops exert distinct effects on rhizosphere soil nutrients, microbial diversity, and community structure in sugar beet, collectively influencing crop growth. Compared to sunflower and corn stubbles, oat and potato stubbles significantly enhanced the organic matter content, available nitrogen, phosphorus, and potassium levels, as well as alkaline phosphatase, urease, and sucrase activities in the sugar beet rhizosphere soil. These improvements were associated with significantly greater dry matter accumulation, yield, and sugar yield in sugar beet. Additionally, bacterial diversity was notably lower under corn, oat, and potato stubbles than under sunflower stubble, whereas fungal diversity was significantly reduced in sunflower stubble compared to corn, oat and potato stubbles. Across all treatments, the dominant bacterial phyla in sugar beet rhizosphere soil were Firmicutes and Acidobacteriota, while the predominant fungal phyla were Ascomycota and Mortierellomycota. Notably, sunflower stubble had a more pronounced effect on the relative abundance of dominant bacterial and fungal phyla than corn, oat, or potato stubbles. These findings support the initial hypothesis that preceding crops shape the structure and function of the sugar beet rhizosphere microbiome by selecting for specific microbial taxa, thereby enhancing soil nutrient availability through microbial community modulation.

## Data Availability

The datasets presented in this study can be found in online repositories. The names of the repository/repositories and accession number(s) can be found in the article/**Supplementary Material**.

## References

[B1] Adekunle A. S. Segun A. A. Oluranti B. O. (2021). Metagenomic insight into the community structure of maize-rhizosphere bacteria as predicted by different environmental factors and their functioning within plant proximity. Microorganisms 9, 1419–1419. doi: 10.3390/microorganisms9071419, PMID: 34209383 PMC8304108

[B2] Borase D. N. Nath C. P. Hazra K. K. Senthilkumar M. Singh S. S. Praharaj C. S.. (2020). Long-term impact of diversified crop rotations and nutrient management practices on soil microbial functions and soil enzymes activity. Ecol. Indic. 114, 106322. doi: 10.1016/j.ecolind.2020.106322

[B3] Chhikara N. Kushwaha K. Sharma P. Gat Y. Panghal A. (2019). Bioactive compounds of beetroot and utilization in food processing industry: Acritical review. Food Chem. 272, 192–200. doi: 10.1016/j.foodchem.2018.08.022, PMID: 30309532

[B4] Chichongue Ó. Van Tol J. J. Ceronio G. M. Du Preez C. C. Kotzé E. (2022). Short-term effects of tillage systems, fertilization, and cropping patterns on soil chemical properties and maize yields in a loamy sand soil in Southern Mozambique. Agronomy 12, 1534. doi: 10.3390/agronomy12071534

[B5] De Chaves M. G. Silva G. G. Z. Rossetto R. Edwards R. A. Tsai S. M. Navarrete A. A. (2019). Acidobacteria subgroups and their metabolic potential for carbon degradation in sugarcane soil amended with vinasse and nitrogen fertilizers. Front. Microbiol. 10. doi: 10.3389/fmicb.2019.01680, PMID: 31417506 PMC6682628

[B6] De Graaff M. A. Classen A. T. Castro H. F. SChadt C. W. (2010). Labile soil carbon inputs mediate the soil microbial community composition and plant residue decomposition rates. New Phytol. 188, 1055–1064. doi: 10.1111/j.1469-8137.2010.03427.x, PMID: 21058948

[B7] Deihimfard R. Sajjad R. M. Chenu K. (2019). Risk assessment of frost damage to sugar beet simulated under cold and semiarid environments. Int. J. Biometeorol. 63, 511–521. doi: 10.1007/s00484-019-01682-5, PMID: 30756175

[B8] Geng G. Li R. R. Stevanato P. Lv C. H. Lu Z. Y. Yu L. H.. (2020). Physiological and transcriptome analysis of sugar beet reveals different mechanisms of response to neutral salt and alkaline salt stresses. Front. Plant Sci. 11. doi: 10.3389/fpls.2020.571864, PMID: 33193507 PMC7604294

[B9] Geng G. Yang J. (2015). Sugar beet production and industry in China. Sugar Tech. 17, 13–21. doi: 10.1007/s12355-014-0353-y

[B10] Guo X. J. Yang J. C. Feng X. J. Jiang C. (2016). Effects of different previous crops in rotation system on dry matter accumulation, quality, and yield in flax. Crops 2, 165–167. doi: 10.16035/j.issn.1001-7283.2016.02.030

[B11] He B. Xue C. Sun Z. Ji Q. Wei J. Ma W. (2022). Effect of different long-term potassium dosages on crop yield and potassium use efficiency in the maize-wheat rotation system. Agronomy 12, 2565. doi: 10.3390/gronomy12102565

[B12] Holmquist L. Dölfors F. Fogelqvist J. Cohn J. Kraft T. Dixelius C. (2021). Major latex protein-like encoding genes contribute to Rhizoctonia solani defense responses in sugar beet. Mol. Genet. Genomics 296, 155–164. doi: 10.1007/s00438-020-01735-0, PMID: 33118051 PMC7840631

[B13] Huang W. J. Sun D. L. Wang R. H. An Y. X. (2021). Integration of transcriptomics and metabolomics reveals the responses of sugar beet to continuous cropping obstacle. Front. Plant Sci. 12. doi: 10.3389/fpls.2021.711333, PMID: 34777408 PMC8578061

[B14] Huang W. Sun D. Fu J. Zhao H. Wang R. An. Y. (2020). Effects of continuous sugar beet cropping on rhizospheric microbial communities. Genes 11 (1): 13. doi: 10.3390/genes11010013, PMID: 31877827 PMC7017100

[B15] Ji R. Q. Xu Y. Si Y. J. Phukhamsakda C. Li Y. Meng L. P.. (2022). Fungal-bacterial networks in the habitat of SongRong (*Tricholoma matsutake*) and driving factors of their distribution rules. J. Fungi 8, 575. doi: 10.3390/jof8060575, PMID: 35736058 PMC9225054

[B16] Kang Y. C. Liu Y. Qin S. H. Zhang W. N. Shi M. F. Fan Y. L.. (2020). Ridge-mulch tillage and rotation with broad bean affects soil microbial community, diversity and crop yield in a long-term potato continuous cropping field. Soil Use Manag. 37, 677–688. doi: 10.1111/sum.12628

[B17] Li X. Z. Rui J. P. Mao Y. J. Yannarell A. Mackie R. (2014). Dynamics of the bacterial community structure in the rhizosphere of a maize cultivar. Soil Biol. Biochem. 68, 392–401. doi: 10.1016/j.soilbio.2013.10.017

[B18] Li M. Yang F. Z. Wu X. Y. Yan H. Liu Y. (2020). Effects of continuous cropping of sugar beet (*Beta vulgaris L.*) on its endophytic and soil bacterial community by high-throughput sequencing. Ann. Microbiol. 70, 39. doi: 10.1186/s13213-020-01583-1588

[B19] Liu R. H. Pan Y. F. Bao H. Liang S. C. Jiang Y. Tu H. R.. (2020). Variations in soil physico-chemical properties along slope position gradient in secondary vegetation of the Hilly Region, Guilin, Southwest China. Sustainability 12, 1303. doi: 10.3390/su12041303

[B20] Mall A. K. Misra V. Pathak A. D. Srivastava S. (2021). Sugar beet cultivation in India: Prospects for bio-ethanol production and value-added co-products. Sugar Tech. 23, 11–17. doi: 10.1007/s12355-021-01007-1000, PMID: 34248307 PMC8261398

[B21] Nabi F. Yang G. T. Sajid S. Chen H. Kaleri A. R. Chen T.. (2022). Linking soil microbial community with the changes in soil physicochemical properties in response to long-term agricultural land use change of different chronosequences and depth layers. Ecol. Indic. 145, 109727. doi: 10.1016/j.ecolind.2022.109727

[B22] Qiao Y. Z. Wang T. T. Huang Q. W. Guo H. Y. Zhang H. Xu Q. C.. (2024). Core species impact plant health by enhancing soil microbial cooperation and network complexity during community coalescence. Soil Biol. Biochem. 188, 109231. doi: 10.1016/j.soilbio.2023.109231

[B23] Qin S. Yeboah S. Cao L. Zhang J. Shi S. Liu Y. (2017). Breaking continuous potato cropping with legumes improves soil microbial communities, enzyme activities and tuber yield. PloS One 12, e0175934. doi: 10.1371/journal.pone.0175934, PMID: 28463981 PMC5413038

[B24] Trinchera A. Migliore M. Raffa D. W. Ommeslag S. Debode J. Shanmugam S.. (2022). Can multi-cropping affect soil microbial stoichiometry and functional diversity, decreasing potential soil-borne pathogens? A study on European organic vegetable cropping systems. Front. Plant Sci. 13. doi: 10.3389/fpls.2022.952910, PMID: 36237499 PMC9552534

[B25] Vaziritabar Y. Frei M. Yan F. Vaziritabar Y. Honermeier B. (2024). Enhancing nitrogen use efficiency and plant productivity in long-term precrop/crop rotation and fertilization management. Field Crops Res. 306, 109210. doi: 10.1016/j.fcr.2023.109210

[B26] Wang Y. Shi M. F. Zhang R. Y. Zhang W. N. Liu Y. H. Sun D. X.. (2023). Legume-potato rotation affects soil physicochemical properties, enzyme activity, and rhizosphere metabolism in continuous potato cropping. Chem. Biol. Technol. Agric. 10, 132. doi: 10.1186/s40538-023-00508-2

[B27] Wu X. Zhang T. Zhao J. N. Wang L. L. Yang D. L. Li G.. (2020). Variation of soil bacterial and fungal communities from fluvo-aquic soil under chemical fertilizer reduction combined with organic materials in North China plain. J. Soil Sci. Plant Nutr. 21, 349–363. doi: 10.1007/s42729-020-00365-0

[B28] Wulanningtyas H. S. Gong Y. T. Li P. R. Sakagami N. Nishiwaki J. Komatsuzaki M. (2021). A cover crop and no-tillage system for enhancing soil health by increasing soil organic matter in soybean cultivation. Soil Tillage Res. 205, 104749. doi: 10.1016/j.still.2020.104749

[B29] Xi H. Zhang X. K. Qu Z. Yang D. Y. Alariqi M. Yang Z. G.. (2021). Effects of cotton-maize rotation on soil microbiome structure. Mol. Plant Pathol. 22, 673–682. doi: 10.1111/mpp.13053, PMID: 33774915 PMC8126184

[B30] Xu F. D. Li C. Chen Y. X. Wu J. C. Bai H. D. Fan S. G.. (2024). Soil microbial community structure and soil fertility jointly regulate soil microbial residue carbon during the conversion from subtropical primary forest to plantations. Geoderma 441, 116767. doi: 10.1016/j.geoderma.2023.116767

[B31] Yan J. Y. Ren T. Wang K. K. Ye T. H. Song Y. Cong R. H.. (2023). Optimizing phosphate fertilizer input to reduce phosphorus loss in rice-oilseed rape rotation. Environ. Sci. pollut. Res. 30, 31533–31545. doi: 10.1007/s11356-022-24133-y, PMID: 36449245

[B32] Yin C. T. Larson M. Lahr N. Paulitz T. (2023). Wheat rhizosphere-derived bacteria protect soybean from soilborne diseases. Plant Dis. 108, 1565–1576. doi: 10.1094/PDIS-08-23-1713-RE, PMID: 38105448

[B33] Zhang Y. P. Li W. Lu P. Xu T. Y. Pan K. (2022). Three preceding crops increased the yield of and inhibited clubroot disease in continuously monocropped Chinese cabbage by regulating the soil properties and rhizosphere microbial community. Microorganisms 10, 799. doi: 10.3390/microorganisms10040799, PMID: 35456849 PMC9028536

[B34] Zhang Y. Li Q. W. Zhang G. F. (2014). Effects of stubble and sowing date on dry matter accumulation and filling of wheat with different muscle types. Zhejiang Agric. Sci. 9, 1343–1346. doi: 10.16178/j.issn.0528-9017.2014.09.070

[B35] Zhang H. F. Luo G. W. Wang Y. Z. Fei J. C. Rong X. M. Peng J. W.. (2023). Croprotation-driven change in physicochemical properties regulates microbial diversity, dominant components, and community complexity in paddy soils. Agric. Ecosyst. Environ. 343, 108278. doi: 10.1016/j.agee.2022.108278

[B36] Zhao P. Y. Zhou H. F. Liao X. L. Zhao L. F. Zheng Y. X. Xiong T. E.. (2025). The regulation of tobacco growth under preceding crop planting: insights from soil quality, microbial communities, and metabolic profiling. Front. Plant Sci. 16. doi: 10.3389/fpls.2025.1530324, PMID: 39990714 PMC11842363

